# Factors affecting well-being in brain tumor patients: An LMIC perspective

**DOI:** 10.3389/fpsyg.2023.1117967

**Published:** 2023-03-31

**Authors:** Nasim Zahid Shah, Taleaa Masroor, Nida Zahid, Wajeeha Zahid, Aneesa Hassan, Iqbal Azam, Khabir Ahmad, Shireen Shehzad Bhamani, Adnan Abdul Jabbar, Nargis Asad, Muhammad Shahzad Shamim, Rashid Jooma Khan, Gohar Javed, Ehsan Bari, Syed Ather Enam

**Affiliations:** ^1^School of Nursing and Midwifery, Aga Khan University Hospital, Karachi, Pakistan; ^2^Department of Surgery, Aga Khan University Hospital, Karachi, Pakistan; ^3^Department of Community Health Sciences, Aga Khan University Hospital, Karachi, Pakistan; ^4^Department of Oncology, Aga Khan University Hospital, Karachi, Pakistan; ^5^Department of Psychiatry, Aga Khan University Hospital, Karachi, Pakistan

**Keywords:** qualitative research, brain tumor, resilience, quality of life, wellbeing

## Abstract

**Introduction:**

Brain tumor is a devastating and often fatal diagnosis; quality of life and patient well-being are important goals of treatment. This study addresses the gap in culture-specific literature exploring the needs and coping strategies of brain tumor patients within an LMIC setting.

**Methodology:**

A qualitative approach was undertaken using an exploratory descriptive study design. In-depth interviews were conducted to capture the perspective of 250 brain tumor patients at a private tertiary center followed by extensive content analysis to identify major themes and sub-themes across responses.

**Results:**

The analysis identified three major themes: (i) Factors affecting the lives of brain tumor survivors (BTSs) and their impact (ii) What works to improve QoL according to the survivors’ perspectives, and (iii) Coping tactics & fostering healthy relationships. The need for financial navigation strategies improved patient-physician relationships, and reinforcing positive coping strategies were emphasized.

**Conclusion:**

In our population, family support and spiritual connection played an important role in helping patients mitigate the psychosocial burden of illness. However, financial concerns were pervasive and need to be addressed for better overall well-being.

## Introduction

Globally brain tumor has an annual incidence of 3.5 cases and an annual mortality of 2.8 cases, per population of 100,000 (All Cancers, 2020; Brain, Central Nervous System, 2020), indicating high mortality among those diagnosed. In Pakistan, the mortality rate among brain tumor patients ranges from 60 to 80% with ~150,000 new cases diagnosed each year (Yusuf, 2013). Since most primary brain tumors follow a devastating clinical course, one where the patient progressively deteriorates and eventually dies, improving quality of life and minimizing distress are essential goals of treatment (Kvale et al., 2009).

Conventionally, the focus of oncologic care has been on achieving the medical endpoints of survival, local control, and preventing recurrence. The psychosocial factors though important, are often overlooked during clinical practice. Studies report a high rate of depression and anxiety among brain tumor patients and their caregivers (Janda et al., 2008). A study showed anxiety prevalent in 30% of patients and 40% of carers respectively, and a depression incidence of 17 and 10% respectively, on the Hospital Anxiety and Depression Scale (Janda et al., 2007). These rates are higher than those experienced by patients with other types of cancer and can interfere with treatment compliance and Quality of Life (QoL). Conversely, factors such as resilience can be protective against stressors, presupposing fewer requirements for psychosocial support and leveraging better overall well-being (Janda et al., 2007).

To formulate an effective intervention it is important to understand the culture-specific needs of the target population. We aimed to identify factors that improve the QoL of brain tumor patients within the demographic of a low to middle-income country with a collectivist socio-cultural foundation, specifically focusing on actionable items such as what helps, what harms, and what can maximize well-being.

## Methods

### Study design and setting

This qualitative inquiry was conducted using an exploratory descriptive study design. The study was embedded in the larger analytical cross-sectional survey conducted from November 2019 to May 2020 at the Aga Khan University Hospital (AKUH), Karachi, Pakistan, which is a private-owned, Joint Commission International Accreditation (JCIA) accredited tertiary care hospital in Pakistan and caters to patients from all socioeconomic backgrounds. Though most patients seeking treatment at AKUH are from affording sociodemographic backgrounds, patient welfare programs ensure that low-income patients are provided with some degree of financial support. The purpose of pairing qualitative and quantitative components within this survey was to provide an in-depth understanding of the factors that have affected the lives of brain tumor (BT) patients and also to examine the different coping tactics used by the patients and their families to combat the disease.

In addition, the results obtained by adopting this approach assists in increasing the reliability and credibility of the findings, the methodological triangulation.

Approval for this study was received from the institutional review board of AKUH (Reference Number: 5154-Sur-ERC-17). The complete cross-sectional study has been published by the authors (Zahid et al., 2021).

### Participants recruitment and sampling

This study was embedded within a larger study studying resilience and its associated factors in brain tumor patients (Zahid et al., 2021). Eligible patients were recruited for this qualitative research using a purposive sampling technique. The participants in this study were all adult (18 years) cancer patients treated at AKUH for brain tumors who gave written informed consent, were at least 4 weeks into their course of treatment, and had resided in Pakistan for at least the previous 3 months (Zahid et al., 2021). This study sought to examine the perspective on care and quality of life among brain tumor patients in the context of Pakistan, as patients living abroad are probably exposed to different cultural and social structures. Patients with any known psychiatric illness history, those taking prescription antidepressants, and those suffering from incapacitating comorbidities like stroke or renal failure were all disqualified. Due to the high prevalence of these conditions, patients with hypertension (HTN), diabetes mellitus (T2DM), or chronic obstructive pulmonary disease (COPD) were not excluded. Out of 255 participants who were initially approached, 1 was lost to follow-up and 4 had a history of psychiatric illness. Data for 250 patients were eventually analyzed.

### Data collection

#### Personnel

The in-depth interviews were conducted by trained research assistants (RAs), who were well-versed in the context and local language. The RAs were trained by the principal investigator (PI) on the content and pedagogy for conducting the interviews. All the interviews were conducted in the national language (Urdu) to maintain the comfort level of the patients and to gain an in-depth understanding of the factors related to the disease under discussion.

#### Interview guide

A semi-structured interview guide was developed to conduct in-depth interviews with the patients. The interview guide was developed based on an extensive literature review on the mental and emotional well-being of cancer patients tailored according to our research question, along with the consultation of experts within the research team.

The interview questions were prepared to assess 4 main domains: (i) experiences with BT diagnosis, treatment, and its overall impact on quality of life; (ii) perceptions about factors affecting the quality of life of BT patients; (iii) the impact of disease on their relationship with family members/caregivers, coping mechanisms support received/needed; and (iii) recommendations for promoting strong and healthy relationships within the family dynamics. Example questions included the following:

- Thinking about the course of your disease process, what are the important factors that affect the life of patients suffering from BT?
- Based on your experience, what are the different coping tactics that are used by the patients or their family members to combat the disease?
- After the diagnosis of your disease, do you think of any change in your relationship with your family members? How this condition has changed your caregiver’s relationship?
- In your opinion, what are the ways to foster or promote a healthy and strong relationship with your family members?

Qualitative measurements of the patient’s perceptions regarding the effects of disease on their quality of life, relationship with family members/caregivers, and coping tactics can help to explain and interpret the quantitative measurements: resilience in BT patients and its relationship with patients’ sociodemographic factors, clinical characteristics, social support, and mental health (Zahid et al., 2021).

#### Interviews

The in-depth interviews (IDI) were conducted to capture the perspective of 250 BT patients visiting surgical/oncology clinics at the AKUH. The participants were approached face-to-face. The interviews were conducted by trained female research assistants. Both research assistants were locals, who were well-versed in language and context, therefore were able to build a good rapport with the study participants. Patients were informed that both research assistants had good background knowledge of the research being conducted and had prior experience in conducting in-depth interviews. The interviewers’ relevance and interest in the research topic (having a background in psychology and working with vulnerable groups) were also reported. To maintain confidentiality and privacy, all interviews were held in quiet and private spaces at timings suggested by the patients and each lasted for 30–40 min, with no one but the interviewer and the participant in the room. All the interviews were manually recorded and transcribed verbatim. At the end of each interview, the participants were asked if they wanted to add anything. The transcripts were retained by the interviewer.

### Data analysis

A manual data analysis process was conducted using content analysis (Zahid et al., 2021). The transcripts were independently read by two analysts, well versed in the context and content of the interviews, and they manually developed codes maintaining the manifest and latent meaning of the transcripts. The coding was done by two coders independently followed by cross-checking and consensus on similar categories. Similar codes were merged and the subsequent relevant categories were linked to the different themes. Discussions were held among the team during the process, and a consensus on discrepancies was reached on the overall analysis. Finally, the transcripts were thoroughly reviewed to incorporate the relevant information in the final analysis.

To maintain confidentiality, the names of the patients were kept anonymous and each transcript was given a unique identifier. The data was kept under lock and key and accessible only to the research and audit team.

## Results

### Participant demographics

Out of the 250 participants who were eventually included in the study, 67.6% of our respondents were male and 32.4% were female. 91.6% had received some form of formal schooling and 69% had a monthly household income between the range of 215-430 USD. 82% of our respondents were married, however, there was a good divide between those living in nuclear (46%) and extended-family (54%) setups. A small minority of 28% had spouses who were working also. Heterogeneity was seen in ethnicity as our participants spoke different languages (Urdu, Sindhi, Punjabi, Pushto, Siraiki, Balochi, and Hindko, among others) indicative of their varied backgrounds.

In total, 22.8% of patients said they had had surgery before (other than brain surgery). While 16% of participants had a family history of another cancer, only 4.8% of participants had a family history of brain tumors. Only 4% of the respondents had a history of depression in their families. In the preceding 6 months, 8% of the respondents stated having experienced a death in their family (Zahid et al., 2021).

Gliomas and meningiomas were the most prevalent types of brain cancers, accounting for 46.8 and 21.2%, respectively, and 78% of participants had undergone tumor biopsies. 60.8% of patients reported no adjuvant therapy, 4.4% reported chemotherapy, 25.2% reported combination therapy, and 9.6% reported receiving radiotherapy (Table 1; Zahid et al., 2021).

**Table 1:** Tumor characteristics.

Tumor characteristics	Frequency (*n*)
Tumor type	
Glioma	117 (46.8)
Meningioma	53 (21.2)
Schwannoma	12 (4.8)
Pituitary	44 (17.6)
Others	24 (9.6)
Surgical intervention	
Only biopsy	195 (78.0)
Only total resection	11 (4.4)
Multiple interventions	27 (10.8)
No surgical intervention	17 (6.8)
Adjuvant therapy	
Chemotherapy	11 (4.4)
Radiotherapy	24 (9.6)
Combination	63 (25.2)
No adjuvant therapy	152 (60.8)
Treatment stage for brain tumor	
On-going	138 (55.2)
Complete	112 (44.8)
Feeding tube needed	2 (0.8)
Tracheostomy needed	1 (0.4)
Urine bag needed	5 (2.0)

### Thematic analysis

This qualitative assessment tends to highlight the experiences and perceptions of brain tumor survivors (BTSs) in Pakistan, under three main themes. The detailed results are regarding the life consequences, ways to improve quality of life, and coping mechanisms.

#### Factors affecting the lives of BTSs and the impact

In general, the participants strongly believe that being diagnosed with BT takes a toll on the patient and their family physically, psychologically, financially, and socially.

##### Impact on physical life

Regarding the impact of BT diagnosis and treatment on physical life, generally, the participants shared that their day-to-day functioning is disturbed as they feel less strength in their bodies to do the tasks. Discussing the underlying symptoms, fatigue, dizziness, body pain and headache were the frequently reported physical issues experienced by the BTSs. Additionally, walking and travel are difficult due to pain in the legs. Few participants mentioned other symptoms including; weight loss, blurry or impaired vision, hair fall, sleep and eating disturbances, nausea, constipation, and seizures.

Moreover, a few others added that due to the non-availability of qualified physicians and appropriate treatment, life became challenging for them as they had to travel from one place to another to receive quality treatment.

“I feel a lot has changed in my life since the diagnosis of my BT. I do not show the strength and courage anymore that I used to have before my illness.” IDI F120

“I feel that I have become slow in everything that I do. I have developed pain in my legs after the operation and walking is painful. I want this pain to subside soon” IDI F40

“There is weakness and headache. Due to weakness, I can’t walk and now I have been forgetting things frequently” IDI F58

“Initially I suffered a lot due to the negligence of doctors in Punjab. I was put on antiepileptic drugs. Finding a good doctor is a true blessing and choose the doctor wisely. Life is upside down if there is no appropriate treatment” IDI M48

“I feel weakness in my left arm, and have less strength in it compared to my right arm. I watch TV but I am unable to concentrate. I feel that I have developed memory loss and have now become more aggressive” IDI M31

##### Influence on psychological wellbeing

With respect to psychological well-being the participants agreed that, though the disease is life-threatening, the word “cancer” has a greater psychological impact than the disease itself. A substantial proportion of participants expressed that they often feel depressed, and worried, and loss of productivity becomes a routine in their daily activities. Additionally, others explained that these originated from the fear of disease progression/reoccurrence and helplessness. Some BTSs felt depressed and worried about their future as they perceived themselves as less capable than others of fulfilling their dreams (career and education). They believe that due to this disease they are not able to do things with freedom as they used to do before. Finally, a few of the respondents reported aggression (sometimes taking it out on children), intolerance, and becoming superstitious, after their diagnosis and treatment.

“This word sartaan [meaning cancer in local dialect] or cancer is very bad. The fear of this word has a greater impact on the patient than the disease itself” IDI F77

“I often feel depressed. There is discomfort and dislike for life and my surroundings” IDI F88

“I feel completely broken. My education has been totally ruined because of my illness. I want to opt for my studies” IDI M67

Some of the BTSs on the contrary reported a sound, optimistic and resilient attitude while describing their journey. They agreed that the influence and impact of the disease differ from patient to patient as it depends upon their willpower and resilience. If the individual has strong willpower and greater resilience, then the condition will have a lesser impact. Adding to the discussion few mentioned that they have become more caring toward their health as they try to face the situation with courage, happiness, and positive thinking.

“I think I have no such effects on my life due to my disease, as I never felt that I have been diagnosed with BT. If your willpower is weak then anything will impact you, so one should work to have strong willpower”. IDI F73

##### Financial impact of BT diagnosis and treatment

Throughout interviews, participants frequently shed light on the financial impact of BT treatment on themselves and their families. They voiced frustrations over the arrangement of finances to bear the treatment and travel costs involved. Additionally, they expressed their stress about the non-availability of financial assistance programs/schemes by the authorities for patients in need (single/retired/non affording patients). For nearly every participant, the financial burden was causing them to feel a fair amount of anxiety and unease.

Moreover, for male participants, being diagnosed with BT has serious impacts on their employment and earnings. They (few being the earning members) reported either losing their employment or not being able to perform job requirements due to generalized weakness and loss of memory and impaired vision. Additionally, a few young male participants shared the discontinuation of studies due to the BT.

“Due to my illness, I feel to have a weak memory now, which has impacted my job as I am not able to do the work required in the job. I am the only earning member, my parents and siblings live in India. I wish to improve my relations with my brothers” IDI M27

Very few participants on the other hand expressed “gratitude” for being able to bear the finances successfully for their treatment but reported their concerns for others who are struggling with financial arrangements.

“Patient becomes helpless since the cost of treatment is very high and receiving timely treatment becomes difficult as the disease progresses. Lack of money and costly treatment has its own impact on patient and family” IDI F142

“The treatment is very expensive. Despite earning a sound income, I am also struggling with managing my expenses. Since the treatment is long-term and expensive, now I am hand to mouth” IDI M141

##### Impacts on family and relationships

The findings concerning the impact of BT diagnosis on any changes in relationships (family, friends & caregivers) were very productive. The majority of participants reported having established a substantially strong family bond during this difficult part of their life. They reported having increased support, love, and compassion from their spouses, children, in-laws, siblings, and parents. Additionally, some male participants also reported having financial support from family and friends for the treatment in addition to their care and concern.

“During the course of my illness, I became very aggressive and touchy. I used to argue and fight over minor issues. But, my family understood my tantrums and irritability. They supported me throughout, especially my husband and children” IDI F166

However, few male participants reported their discomfort with the bad tempers of their relatives during their illness. They shared that such relatives pretend to be kind but internally they [relatives] feel happy for them (patients) being in trouble.

“It hurts the way they [relatives] look at you and talk about you. I don’t like to meet them frequently as they are envious of us (siblings) doing good in our professional lives” IDI M94

#### What works to improve the quality of life of BTSs, survivors’ perspective

With regards to steps taken to improve the quality of life of BTSs, the responses of participants expressed a collective sense of agreement for (i) awareness among patients regarding the disease, (ii) enhanced psychological support, and (iii) effective patient-physician relationship.

##### Patient education and awareness

All the participants strongly urged the need for awareness and information sharing among the patients, regarding the disease, treatment, and progression. They insisted to have counseling and awareness sessions to impart more knowledge, as there is a lack of awareness among common folks. Additionally, it was suggested that physicians should provide awareness and information about treatment modalities other than surgical interventions.

“The doctors should provide information regarding less invasive, painless and quality treatment options to patients” IDI M66

“There should be information sharing and awareness sessions regarding treatment modalities other than operation and about this disease so that the patient doesn’t feel scared of the disease” IDI F99

##### Psychological support

All the participants agreed on the significance of providing psychological counseling and support to the BTSs and patients. In this regard, they highlighted the substantial role of physicians and family/caregivers/friends. They emphasized that the motivation, hope, and strength that a patient receives from a physician and family imparts a psychological boost to “never give up,” “opt for timely treatment,” “stay positive,” and “live life according to his/her own desires.”

Additionally, it was proposed to develop a mechanism for providing financial assistance to needy patients to reduce their burden and improve their psychological well-being and health.

“The patient should be counseled to stay motivated and positive to fight the disease with strength and never give up” IDI F82

“Don’t make the patient feel tense and provide them with a good environment. Counsel them to have faith and motivation. If the person loses strength, then he/she will start feeling weak and helpless” IDI M109

##### Improved patient-physician communication and relationship

The participants in general expressed their concerns about the patient-physician relationship in terms of communication. They proposed that while talking to the BTSs and patients the physicians should be careful of their choice of words. They highlighted the need for physicians to be more patient, kind, humble, and friendly with patients. Furthermore, during the course of treatment, they should answer the concerns of the patients compassionately and not get annoyed by them.

Additionally, they suggested timely and close follow-ups, and reduce waiting time for patients in clinics. Moreover, they recommended reducing the consultancy charges in the OPD clinics for follow-up visits post-surgery.

“The behavior of a doctor should be nice. They should help and satisfy the patients, be kind and humble while talking to them and resolve their concerns” IDI F155

#### Coping tactics and fostering a healthy relationship

While exploring the coping strategies adopted by the BTSs, mostly the responses described family and social support and the significance of having a strong practice of faith and spiritual well-being.

##### Family support

Having strong family support has emerged as a powerful coping strategy throughout the interviews, exercised by the majority of the BTSs in their lives. The participants shared that during illness, the support from their spouse, children, in-laws, and friends has been exceptional and they were able to cope with their unpleasant and negative emotions effectively. They had ample time to spend in family get-togethers and outings. The immense love, respect, compassion, and care that they received from family and friends enabled them to stay positive and motivated. Additionally, watching comedy movies and TV shows together with family members kept the participants engaged in healthy activities diverting their attention from the disease.

“The families should live together and help the patient financially. They should spend quality time (gatherings and healthy activities) with the patient so that the patient never feels alone. They should boost their strength and encourage them that everything will be fine”. IDI M45

##### Prayers and spirituality

In addition to the family support, some participants expressed that they have become more religious and developed a closeness with the creator (GOD and BHAGWAAN). They believe that after being diagnosed with BT they have become regular in their prayers, submissions, and recitations. They strongly believe that detachment from the practice of faith is one of the underlying factors that people get exposed to challenges. Additionally, expression of gratitude and being grateful to the creator brings peace and courage to any life situation.

“After being diagnosed with BT I have become closer to my practice of faith as I regularly recall my GOD and do my holy recitations. Remembering GOD and expressing our gratitude brings peace in life” IDI M69

## Discussion

This exploratory study addresses the extant gap in literature on the experiences of brain tumor patients in an LMIC, and more narrowly, the culturally collectivist Pakistani population. In our study, the role of family and spiritual connection was found to be central to coping with the illness. Strategies to mitigate financial strain, introducing psychosocial interventions, and improving communication between the patient and practitioner are important for improved overall well-being.

Financial constraint is a huge burden for most families in Pakistan. The average monthly income for a middle-class household is ~230 USD (Catt et al., 2011) and the mean monthly cost per patient for cancer care at a private tertiary care hospital is more than 5 times the amount, with the majority of the expenses being borne by the patient’s family (Giovagnoli et al., 2005). The difference between household income and expenditure means optimal care is only attainable with great difficulty. In our study cohort, household income ranged from 187 to 623 USD, with 50.4% having more than 6 members to support (Zahid et al., 2021). During the interviews, nearly all our patients reported financial constraints attributable to treatment costs. Few mentioned becoming “hand-to-mouth” since the start of treatment, and well-to-do patients expressed their concern about the exorbitant expenses. Financial toxicity affects well-being and patients’ ability to opt for the best treatment option (Zaidi et al., 2012). Patients resort to selling assets, using their savings, and utilizing other means to bridge the deficit. A study done at a private tertiary care hospital in Pakistan showed that 34.3% of patients undergoing cancer treatment took loans to pay for their treatment (Giovagnoli et al., 2005). To assist patients financially, Patient Assistance Programs (PAPs) can be created at the institutional level, that draw in support from multiple sponsors and provide aid based on the financial needs of a patient (Sherman and Fessele, 2019). Initiatives at the government level, such as the Health card system introduced by the Punjab government, are a dignified and swift means to reduce out-of-pocket expenditure for the underprivileged and to improve their access (Impact of Trained, 2022).

Most brain tumors follow an aggressive clinical course, one of rapid physical and intellectual decline (Brown et al., 2006; Zahid et al., 2021). Changes in functionality make patients feel less in control (Janda et al., 2008), lowers mood (Creswell and Creswell, 2017), and can reduce their ability to integrate socially (Ford et al., 2012). Our patients reported the desire for better pain management and physical rehabilitation. This conforms with an Australian study that identified physical assistance as an unmet need among cancer patients and high demand for support services (Janda et al., 2008). This need increases in proportion to emotional distress being experienced by both patients and caregivers (Janda et al., 2006, 2008). Furthermore, social decline ensues as an unfortunate by-product, worsening feelings of isolation and distress among patients (Arnold et al., 2008; Cavers et al., 2012; Mccutchan et al., 2021). Our study participants felt very strongly about the role of their friends and family in fostering hope and helping them fight negative feelings associated with their illness. The role of the family in the particular context of our population is pivotal, as it is the norm to live in multi-generational setups and joint families. This provides patients with a strong network of caretakers within the family from whom to leverage emotional, financial, and physical support in times of need (Gouzman et al., 2015).

Diaz et al., hypothesized that depression and anxiety among cancer patients may not be caused by the symptoms but by their perceived level of threat (Kilbride et al., 2007; Cubis et al., 2017). Our findings showed that patients who reported a negative self-image, felt incapable of achieving their dreams, and were anxious about the recurrence or worsening of the disease, were more likely to report symptoms of depression, anxiety, and unproductiveness. Conversely, patients who felt more in control over their lives and who associated the impact of disease with internal factors such as willpower and resilience were generally optimistic and likely to interpret their experience more positively. They saw cancer as an opportunity to become more “caring toward their health.” One even reported not having ever felt like a brain tumor patient, followed by: “*If your willpower is weak then anything will impact you, so one should work to have strong willpower.” IDI F73.*

Another important factor reported by patients as something likely to benefit them was a healthy patient-practitioner relationship. An approachable manner, encouragement, and good communication are crucial to improving patient experience. Information about the duration, symptoms, and side effects of treatment can reduce anxiety (Díaz et al., 2009) and enable them to make better decisions. A study done on the impact of proper information during surgical decision-making of patients with high-grade glioma showed that patients who are given information and have better comprehension of the procedure, experience less anxiety than those who are not (Kilbride et al., 2007). Patients also emphasized the need for awareness and education on alternative modalities of treatment. This is an important consideration as one of the major reasons for care-seeking delays in LMICs is the utilization of traditional, complementary, or alternative medicine (TCAM; Southam-Gerow et al., 2011).

A person diagnosed with advanced metastatic cancer is changed by the realization of the terminal nature of their illness and is perceived differently by those around them (Arnold et al., 2008). Near end-of-life, physical-wellbeing becomes less important, and existential issues such as the meaning of life come to the fore (McMullen, 2019). These spiritual needs can be religion-specific, or outside the realm of religiosity. In our cohort since the majority ascribed to the Islamic or Hindu faith, spirituality embedded within religion was found to be an important way of coping. The Hindu faith sees death as a process of rebirth for the soul. For Muslims, dying after suffering is seen as honorable, one where all their sins have been washed off and the promise of reward in the afterlife awaits. Both these thoughts can grant peace and a sense of calm to a dying individual, where termination of life only means an end to their physical existence and not their spirit. In conventional medical practice physicians rarely receive training in how to address patients’ spiritual needs (About The Program, 2022). These conversations should be encouraged and inquiries into spiritual well-being should be incorporated into standard practice (Emanuel and Emanuel, 1998).

### Strengths and limitations

Our study is the first to do a qualitative inquiry into brain tumor patients’ (Graneheim and Lundman, 2004) perceptions of their illness, within a Pakistani demographic. This attempt was embedded within a larger study that evaluated quantitative measurements of resilience, anxiety, and depression within the same cohort (Zahid et al., 2021), allowing us to draw connections and establish the plausibility of our data. We used interview responses as our unit of analysis and coded them to draw meaningful conclusions. Categorization of responses into themes and sub-themes allowed us to establish the logical coherency and credibility of our findings. We maintained consistency in our interviewing process by training our interviewers to use the same script and style across patients. Our questionnaire did not undergo any modifications during the study period, increasing the dependability of our results (Graneheim and Lundman, 2004). Through in-depth interviewing, we were able to study the role of family structure, religion, and the patient-practitioner relationship unique to our socio-culture setting, on the overall well-being of patients, helping us to explain the paradoxically low measurements of psychological distress within our population. Most importantly, our study identified the pervasiveness of financial toxicity as a common stressor across the sociodemographic spectrum, highlighting the role of financial support programs and financial navigators in the care setting. Since our study was conducted in a private tertiary care hospital, arguably catering to the affluent rather than the average Pakistani household, the generalizability of our findings is limited. However, the themes we were able to identify can serve as guideposts for optimizing patient care in any healthcare setup catering to brain tumor patients in low to middle-income economies, with a similar socio-culture backdrop.

### Conclusion

Our study offers a unique, intuitive insight into the concerns, challenges, and coping strategies used by brain tumor patients within the Pakistani demographic. Family support and spiritual connection were found to be the most important factors for patients when it came to dealing with the devastating course of their illness. Financial support schemes need to be prioritized in any future initiative to minimize the emotional, financial, and physical distress caused to cancer patients and their families.

## Data availability statement

The original contributions presented in the study are included in the article/supplementary material, further inquiries can be directed to the corresponding author.

## Ethics statement

The studies involving human participants were reviewed and approved by Ethical Review Committee, Aga Khan University. The patients/participants provided their written informed consent to participate in this study.

## Author contributions

NS drafted, analyzed the data, and critically reviewed the manuscript. TM drafted, revised, and critically reviewed the manuscript. NZ conceived the study and critically reviewed the manuscript. WZ and IA critically reviewed the manuscript. AH, KA, SB, and NA overlooked the study. AJ, MS, RK, GJ, EB, and SE were the subject experts and contributed to the design of the study. All authors have contributed intellectually to this manuscript and have read and approved the final manuscript.

## Funding

The work is funded by Aga Khan University’s SEED Money grant award number PF 89-1016.

## Conflict of interest

The authors declare that the research was conducted in the absence of any commercial or financial relationships that could be construed as a potential conflict of interest.

## Publisher’s note

All claims expressed in this article are solely those of the authors and do not necessarily represent those of their affiliated organizations, or those of the publisher, the editors and the reviewers. Any product that may be evaluated in this article, or claim that may be made by its manufacturer, is not guaranteed or endorsed by the publisher.
